# Delineation of Radiation Therapy Target Volumes for Lingual Nerve Involvement

**DOI:** 10.7759/cureus.32993

**Published:** 2022-12-27

**Authors:** Stephen J Sozio, Suresh K Mukherji, Kenneth Hu, Elcin Zan, Anastasia Tjan, Sung Kim

**Affiliations:** 1 Radiology, Rutgers Robert Wood Johnson Medical School, New Brunswick, USA; 2 Radiology, University of Illinois, Peoria, USA; 3 Radiation Oncology, New York University (NYU) Langone Health, New York, USA; 4 Radiology, New York University (NYU) Langone Health, New York, USA; 5 Radiology, Siloam Radiology Indonesia, South Kalimantan, IDN; 6 Radiation Oncology, Rutgers Cancer Institute of New Jersey, New Brunswick, USA

**Keywords:** head and neck radiology, radiation oncology education, lingual nerve, radiation therapy contouring, head and neck neoplasms

## Abstract

It is important for radiation oncologists to be able to accurately contour the lingual nerve pathway, as it is commonly involved in oral cavity cases. However, most atlases do not give a detailed account of the entire lingual nerve pathway as it traverses from the oral cavity, through the masticator space, to the base of the skull. Three experienced head and neck cancer specialists (two radiation oncologists and one neuroradiologist) examined anatomy textbooks, institutional magnetic resonance imaging (MRI), and computed tomography (CT) images of normal anatomy and also recurrences along the lingual nerve pathway to determine “anchor points” to help radiation oncologists contour more confidently. We found five anchor points to help radiation oncologists contour the lingual nerve pathway: At the level of the foramen ovale, the lateral pterygoid, the transition between lateral and medial pterygoid, the medial pterygoid (within the pterygomandibular space), and the oral cavity. Five anchor points with easily identifiable anatomy are established that radiation oncologists can use to contour the lingual nerve pathway more confidently.

## Introduction

The extent of perineural invasion (PNI) can range from microscopic PNI to macroscopic PNI, which refers to the clinical or radiological involvement of larger nerves [1]. Cases of microscopic PNI are typically treated to at least 54 Gy along the nerve course. Macroscopic PNI with negative margins may be treated to 60 Gy along the nerve course, while macroscopic PNI with positive margins should receive 66 Gy to the tumor bed and 60 Gy along the remainder of the nerve course [2].

The lingual nerve provides gustatosensory function to the floor of the mouth, gingiva along the lingual aspect of the mandibular teeth, anterior two-thirds of the tongue, and also provides taste to the anterior two-thirds of the tongue, and parasympathetic innervation to the submandibular and sublingual glands via the chorda tympani [3-6]. The mandibular division of the trigeminal nerve exits the base of the skull through the foramen ovale, then trifurcates into the lingual, auriculotemporal, and inferior alveolar nerves [3,4,6]. Within the infratemporal fossa, the lingual nerve passes medial to the lateral pterygoid muscle (between the tensor veli palatini and lateral pterygoid muscle), travels between the medial and lateral pterygoid muscles, then travels lateral to the medial pterygoid muscle in the pterygomandibular space [6]. It continues anteriorly adjacent to the mandibular ramus towards the oral cavity, ultimately passing under the submandibular duct in its path toward the apex of the tongue [6].

In the setting of oral cavity squamous cell carcinoma (SCC), the incidence of perineural involvement at initial presentation is unclear; however, several studies have estimated its incidence to range as high as 20%-40% [7,8]. For radiation oncologists, knowing how to accurately contour the lingual nerve is essential, as failure along this pathway is often non-salvageable. However, the course of the lingual nerve is not trivial, and while some publications describe contouring from the oral cavity to the foramen ovale, they do not describe how to contour the lingual nerve pathway in between. In this paper, we propose a series of “anchor points” to facilitate contouring the entirety of the lingual nerve pathway.

## Case presentation

Four experienced head and neck cancer specialists (two radiation oncologists and two neuroradiologists) examined anatomy textbooks, institutional magnetic resonance imaging (MRI), and computed tomography (CT) images of normal anatomy and also recurrences along the lingual nerve pathway. During a series of multi-disciplinary conferences, multiple anchor points were established, which are intended to be anatomic locations where radiation oncologists could localize the lingual nerve throughout its course.

We present five anchor points to help localize and contour the lingual nerve pathway at the following levels: 1) foramen ovale, 2) lateral pterygoid muscle, 3) transition between the lateral and medial pterygoid muscles, 4) medial pterygoid muscle, and 5) oral tongue. To illustrate, each anchor point is presented below with a combination of multiple MRI and/or CT imaging of normal anatomy and/or an anatomical design reference.

At the level of the foramen ovale

**Figure 1:**
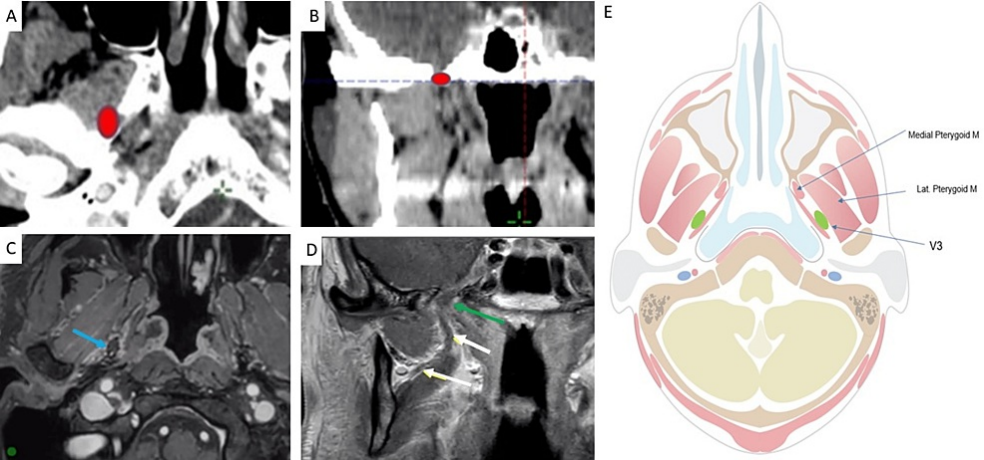
Anchor point at the level of the foramen ovale A: Location of V3 (red) on axial CT B: Location of V3 (red) on corresponding coronal CT slice C: Location of V3 (blue arrow) on axial fat-suppressed contrast-enhanced T1-weighted (T1W) MRI D: Location of foramen ovale (green arrow) and V3 medial to lateral pterygoid muscle (white arrows) on axial fat-suppressed contrast-enhanced T1 MRI E: Anatomic design, created by author Anastasia Tjan

This anchor point is illustrated in Figure 1.

At the level of the lateral pterygoid

**Figure 2:**
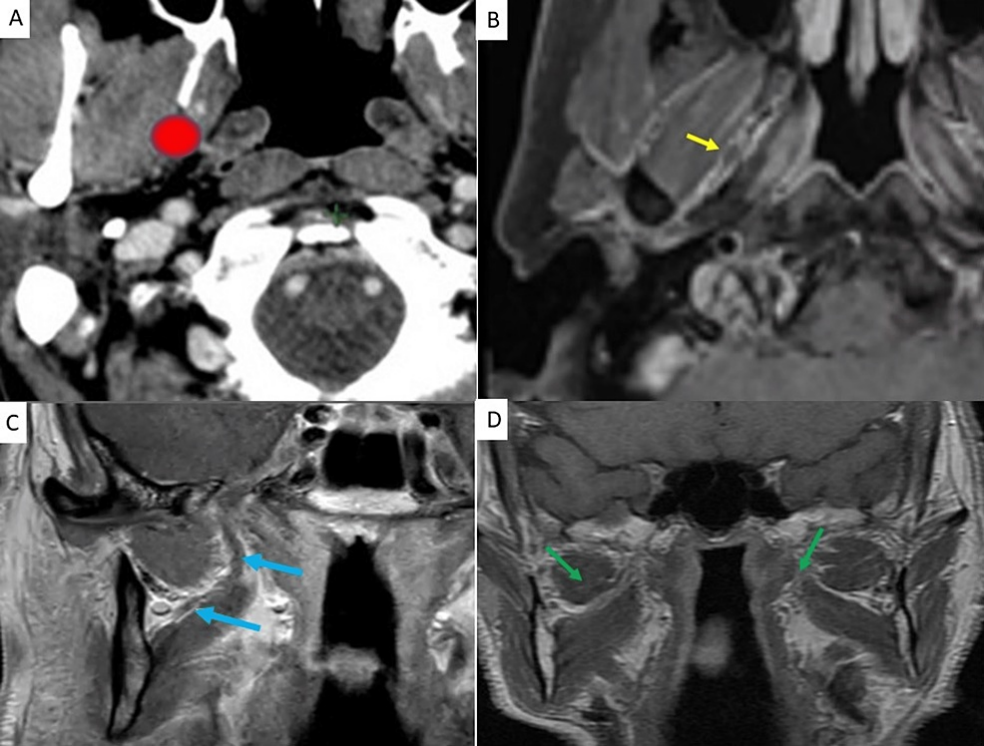
Anchor point at the level of lateral pterygoid A: Location of V3 (red) on axial CT shows how V3 travels medial to the lateral pterygoid muscle and lateral to the tensor veli palatini muscle B: Location of V3 (yellow) on axial fat-suppressed contrast-enhanced T1W MRI C: Location of V3 medial to the lateral pterygoid muscle (blue arrows) on coronal fat-suppressed contrast-enhanced T1 MRI D: Retrograde recurrence along a lingual nerve on contrast-enhanced coronal T1 MRI. The green arrow on the right shows the normal appearance of the left lingual nerve coursing between the medial and lateral pterygoid muscles. Green arrow on the left shows diffuse enhancement and thickening of the right lingual nerve due to perineural tumor spread

As illustrated in Figure 2, upon entering the infratemporal fossa, the lingual nerve passes between the tensor veli palatini and the medial edge of the lateral pterygoid muscles [3]. The lingual nerve and inferior alveolar nerves eventually descend on the medial side of the lateral pterygoid muscle, ultimately separating the superomedial aspect of this muscle [3].

Upon entering the infratemporal fossa, the lingual nerve passes between the tensor veli palatini and the medial edge of the lateral pterygoid muscles [3]. The lingual nerve and inferior alveolar nerves eventually descend on the medial side of the lateral pterygoid muscle, ultimately separating the superomedial aspect of this muscle [3,9].

At the transition between the medial and lateral pterygoid muscles

**Figure 3:**
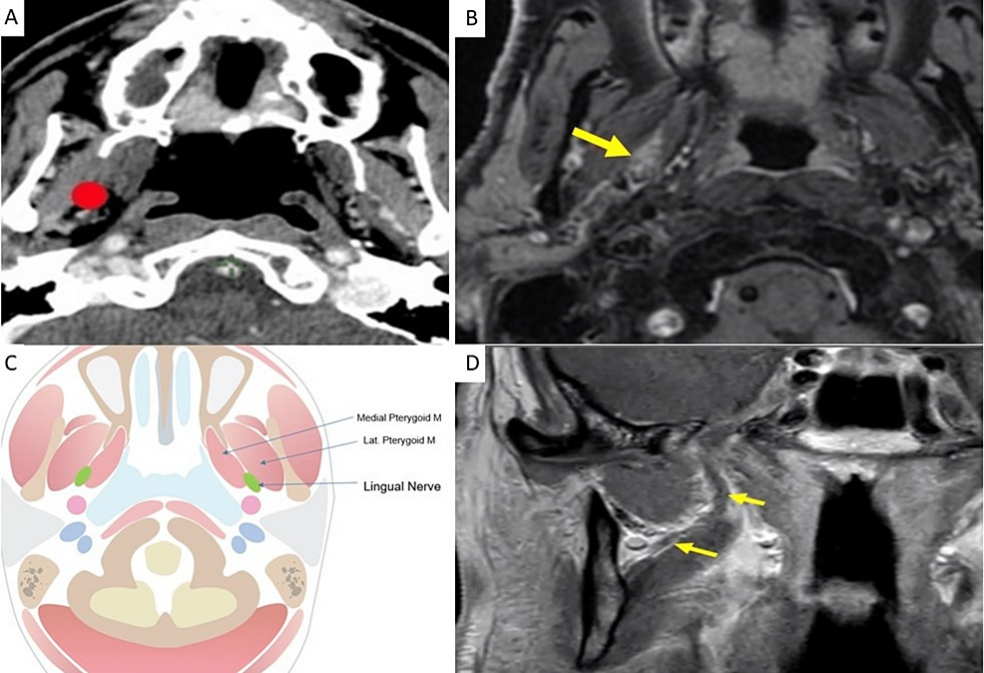
Anchor point at the level of transition between lateral and medial pterygoids A: Location of V3 (red) on axial CT B: Recurrence along a lingual nerve on axial fat-suppressed contrast-enhanced T1W MRI. The yellow arrow shows abnormal enhancement and thickening of the lingual nerve coursing between the medial and lateral pterygoid muscle corresponding to the expected location of the lingual nerve. C: Axial anatomic illustration depicting the course of the lingual nerve between lateral and medial pterygoid muscles, created by author Anastasia Tjan D: Normal appearance of V3 on coronal non-contrast T1W MRI. V3 (yellow arrows) passes between the lateral and medial pterygoid muscles where it divides into the lingual nerve (anterior) and inferior alveolar nerve (posterior)

After passing between the tensor veli palatini and lateral pterygoid muscles, the lingual nerve travels between the medial and lateral pterygoid muscles, as shown in Figure 3 [3]. To more easily contour the transition, we would recommend first contouring at the level of the lateral pterygoid (above) and the medial pterygoid (below), then connecting these contours.

At the level of the medial pterygoid muscle

**Figure 4:**
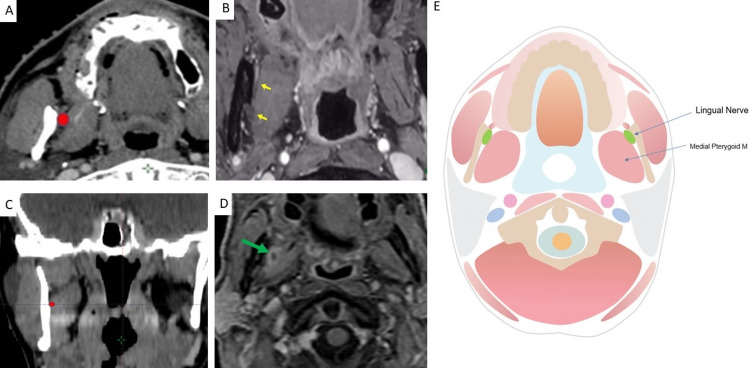
Anchor point at the level of medial pterygoid within the anterior pterygomandibular space A: Location of the lingual nerve (red) in the pterygomandibular space on Axial CT B: Location of the lingual nerve (anterior yellow arrow) relative to the inferior alveolar nerve (posterior yellow arrow), which is entering the mandibular foramen on axial fat-suppressed contrast-enhanced T1W MRI C: Location of the lingual nerve (red) on corresponding coronal CT slice D: Local failure in lingual nerve pathway (green arrow) in pterygomandibular space on axial fat-suppressed contrast-enhanced T1W MRI. Note the position of the enhancing lesion lateral to the medial pterygoid muscle, medial to the mandibular ramus, and anterior to the mandibular notch. E: Anatomic illustration, created by author Anastasia Tjan

As the lingual nerve courses downward, it travels lateral to the medial pterygoid muscle in the pterygomandibular space. [3]. The borders of this space include: 1. anterior (pterygomandibular raphe) 2. posterior (parotid gland) 3. lateral (mandible) 4. medial (medial pterygoid muscle) 5. superior (lateral pterygoid muscle) 6. inferior (inferior mandible). We would recommend contouring the anterior pterygomandibular space, i.e., anterior to the mandibular foramen (since the inferior alveolar nerve enters the mandible via the mandibular foramen, and the lingual nerve travels anterior to the inferior alveolar nerve at this level). This level is illustrated in Figure 4.

At the level of the oral tongue

**Figure 5:**
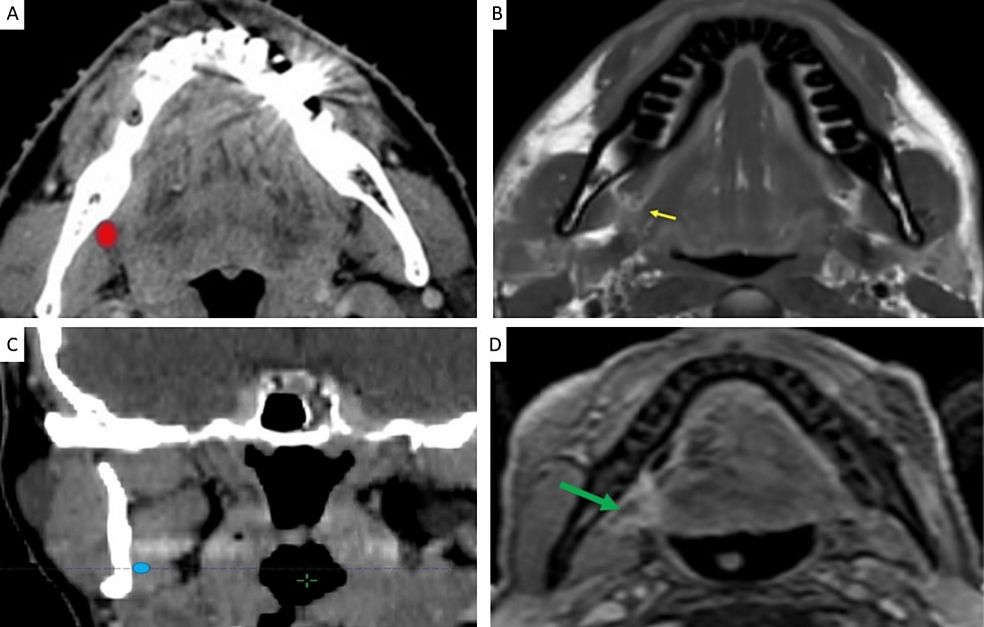
Anchor point at the level of oral tongue A: Location of the lingual nerve (red) located at the inferior pterygomandibular space is located between the anterior margin of the medial pterygoid muscles and the posterior free margin of the mylohyoid muscle B: Location of the lingual nerve (yellow arrow). Axial non-contrast T1W MR shows the lingual nerve located between the anterior margin of the medial pterygoid muscle and the posterior margin of the mylohyoid muscle C: Location of the lingual nerve (blue) on corresponding coronal CT slice D: Local failure in the lingual nerve pathway (green arrow). Fat-suppressed contrast-enhanced T1W sequence shows abnormal enhancement and thickening of the lingual nerve at the expected location

As shown in Figure 5, the lingual nerve enters the mouth by passing beneath the lower border of the superior constrictor muscle and coursing anteroinferior to the lateral surface of the tongue [7]. Next, the nerve then runs along the medial aspect of the retromolar trigone and anteriorly along a superior lingual alveolar crest located on the medial aspect of the third and second molars [7]. Lastly, it turns anteromedially at the posterior attachment of the mylohyoid muscle to the mandible [3].

This is a complicated pathway at this level, but in most cases, as you contour the lingual nerve pathway in the pterygomandibular space to its inferior boundary at the level of the inferior medial pterygoid muscle, the lingual nerve pathway starts to overlap and is subsumed by, the primary site postoperative contour in the oral cavity [7].

Integrated example

**Figure 6:**
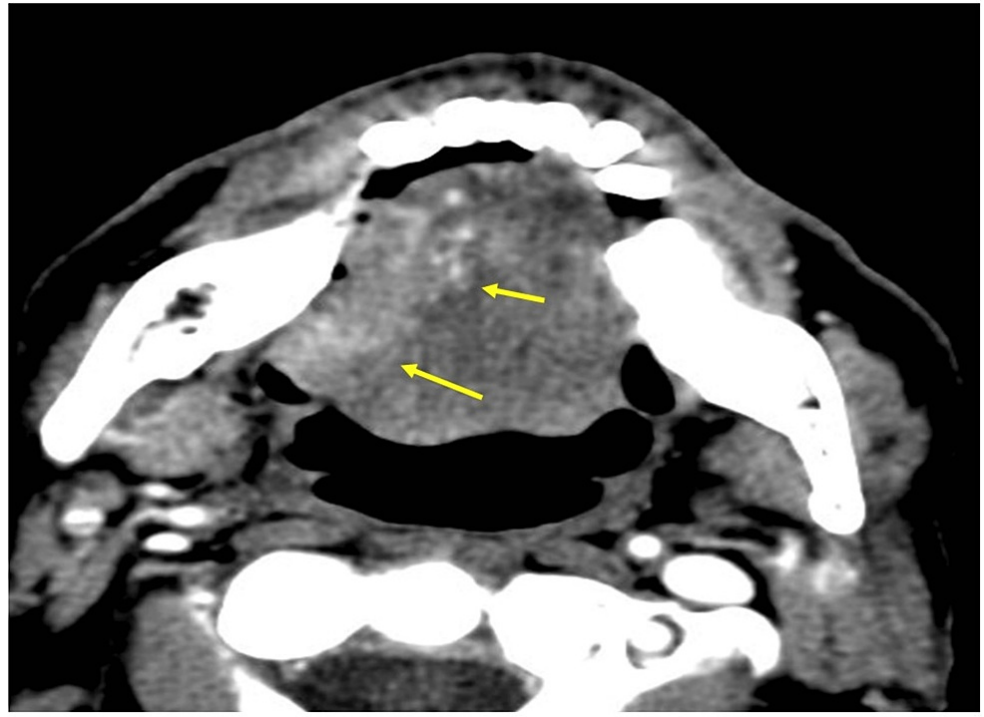
Preoperative contrast-enhanced CT shows a large SCC of the right lateral tongue (yellow arrows)

In the figures below, we present an integrated example to illustrate the use of these anchor points in a single patient. This patient underwent resection of oral tongue SCC, where pathology revealed a 4.2 cm neoplasm with a 1.3 cm depth of invasion and with microscopic PNI of the right lingual nerve. A representative image from the preoperative, contrast-enhanced axial-CT neck is provided in Figure 6.

**Figure 7:**
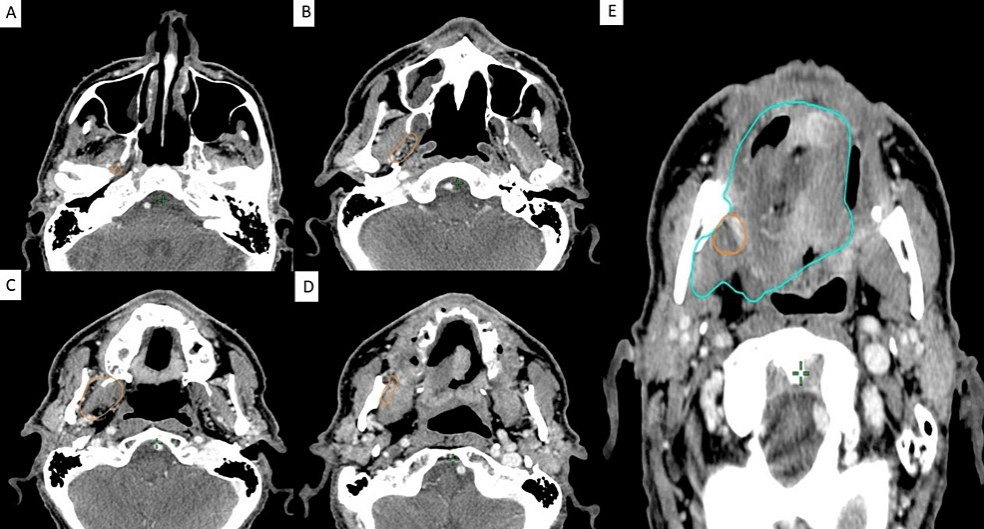
Example of lingual nerve pathway contouring in accordance with anchor points in a postoperative patient A: Clinical target volume of 54 Gy (CTV54) at the level of the foramen ovale B: CTV54 at the level of the lateral pterygoid muscle C: CTV54 at the transition between lateral to medial pterygoid muscle D: CTV54 at the level of medial pterygoid muscle E: CTV54 at the level of the oral cavity (CTV54 contour overlaps with and is subsumed by the clinical target volume of 60 Gy covering primary tumor)

After resection of the mass, the patient was referred for postoperative radiation therapy, with recommended contouring as illustrated in Figure 7.

## Discussion

Perineural invasion (PNI) is defined as tumor cell invasion of the innermost endoneurium, the perineurium surrounding individual nerve fascicles, and/or the outermost epineurium of peripheral nerve, with tumor cells surrounding at least 33% of the total nerve circumference [10]. The process of PNI is driven by multiple molecular pathways in which nerve and cancer cells reciprocally interact to drive the spread of tumors [11]. In contrast to perineural tumor spread (PNTS), in which macroscopic tumor spread can be visualized through direct inspection or relevant imaging study, PNI is a histological finding [11]. Both PNI and PNTS are associated with significantly increased locoregional recurrence and mortality [12]. Radiation therapy (RT) is routinely employed in the treatment of patients suffering from these pathologies to improve overall survival [10,12].

Understanding the anatomy of the lingual nerve pathway is crucial when treating head & neck cancer. Particularly in the setting of PNI, accurately contouring this nerve becomes absolutely crucial, as locoregional failure along this specific pathway results in unsalvageable disease [10]. In keeping, we have tried to make this task less daunting by providing five anatomic anchor points with relevant lingual nerve anatomy and images of locoregional failures to guide and facilitate contouring.

Beyond contouring itself, knowledge of lingual nerve anatomy can raise a radiation oncologist’s vigilance in ruling out lingual nerve PNI. For example, a radiation oncologist seeing a patient who had a large lateralized cancer in the oral tongue/floor of mouth preoperatively (as in Figure 6) should be very mindful that lingual nerve PNI is likely. While the classic teaching is to check the pathology report and contour for PNI only if there is a named nerve, this presupposes a high level of communication between the otolaryngology surgeon and the pathologist. Instead, seeing preoperative imaging like Figure 6, we would recommend that the radiation oncologist query pathology and the otolaryngology surgeon to make sure that PNI does not exist.

## Conclusions

PNI is a histological diagnosis that if not appropriately identified and treated utilizing surgical intervention and/or radiation therapy, results in increased morbidity, mortality, and locoregional recurrence. Specific to the lingual nerve, accurate contouring of its pathway is essential in all circumstances, but especially in the setting of PNI, as failure along this pathway often results in non-salvageable disease recurrence. This nerve's particularly complex course, compounded with distortion of the normal anatomy due to tumor and/or prior surgical intervention, creates an opportunity for key aspects of this nerve to be inadequately contoured in its entirety. As such, it is essential to establish key landmarks that can be used to identify the nerve in its entirety, thus facilitating accurate and complete contouring of the nerve. In this publication, we establish five anchor points, each with easily identifiable anatomy, which can be utilized by radiation oncologists to confidently contour the entirety of the lingual nerve pathway.
